# Detection of sedentary time and bouts using consumer-grade wrist-worn devices: a hidden semi-Markov model

**DOI:** 10.1186/s12874-024-02311-5

**Published:** 2024-09-30

**Authors:** Agus Salim, Christian J. Brakenridge, Dulari Hakamuwa Lekamlage, Erin Howden, Ruth Grigg, Hayley T. Dillon, Howard D. Bondell, Julie A. Simpson, Genevieve N. Healy, Neville Owen, David W. Dunstan, Elisabeth A. H. Winkler

**Affiliations:** 1 Baker Heart & Diabetes Institute, Melbourne, Australia; 2 Centre for Epidemiology and Biostatistics, Melbourne School of Population and Global Health, The University of Melbourne, Melbourne, Australia; 3 School of Mathematics and Statistics, The University of Melbourne, Melbourne, Australia; 4 Active Life Lab, South-Eastern Finland University of Applied Sciences, Mikkeli, Finland; 5 Physical Activity Laboratory, Baker Heart & Diabetes Institute, Melbourne, Australia; 6 Centre for Urban Transitions, Swinburne University of Technology, Melbourne, Australia; 7 Institute for Physical Activity and Nutrition, Deakin University, Melbourne, VIC Australia; 8 School of Human Movement and Nutrition Sciences, The University of Queensland, Brisbane, Australia

**Keywords:** Machine learning, Step counts, Heart rate, Bouts, Wearables data

## Abstract

**Background:**

Wrist-worn data from commercially available devices has potential to characterize sedentary time for research and for clinical and public health applications. We propose a model that utilizes heart rate in addition to step count data to estimate the proportion of time spent being sedentary and the usual length of sedentary bouts.

**Methods:**

We developed and trained two Hidden semi-Markov models, STEPHEN (STEP and Heart ENcoder) and STEPCODE (STEP enCODEr; a steps-only based model) using consumer-grade Fitbit device data from participants under free living conditions, and validated model performance using two external datasets. We used the median absolute percentage error (MDAPE) to measure the accuracy of the proposed models against research-grade activPAL device data as the referent. Bland-Altman plots summarized the individual-level agreement with activPAL.

**Results:**

In OPTIMISE cohort, STEPHEN’s estimates of the proportion of time spent sedentary had significantly (*p* < 0.001) better accuracy (MDAPE [IQR] = 0.15 [0.06–0.25] vs. 0.23 [0.13–0.53)]) and agreement (Bias Mean [SD]=-0.03[0.11] vs. 0.14 [0.11]) than the proprietary software, estimated the usual sedentary bout duration more accurately (MDAPE[IQR] = 0.11[0.06–0.26] vs. 0.42[0.32–0.48]), and had better agreement (Bias Mean [SD] = 3.91[5.67] minutes vs. -11.93[5.07] minutes). With the ALLO-Active dataset, STEPHEN and STEPCODE did not improve the estimation of proportion of time spent sedentary, but STEPHEN estimated usual sedentary bout duration more accurately than the proprietary software (MDAPE[IQR] = 0.19[0.03–0.25] vs. 0.36[0.15–0.48]) and had smaller bias (Bias Mean[SD] = 0.70[8.89] minutes vs. -11.35[9.17] minutes).

**Conclusions:**

STEPHEN can characterize the proportion of time spent being sedentary and usual sedentary bout length. The methodology is available as an open access R package available from https://github.com/limfuxing/stephen/. The package includes trained models, but users have the flexibility to train their own models.

**Supplementary Information:**

The online version contains supplementary material available at 10.1186/s12874-024-02311-5.

## Background

The global sales and use of wearable devices in health care have been increasing and were expected to reach USD 4.5 billion by 2020 [1]. Among these are consumer-grade wrist-worn activity trackers. In addition to providing health-related information to consumers, these devices have also increasingly been used in research settings as both measurement and behavior change tools [2–5]. Wrist-worn devices can be more comfortable to wear and lead to greater adherence and longer wear within the research setting [6, 7], compared to research-grade thigh-worn or hip-worn devices. Their suitability to long-term wear and their widespread use indicates that they may have the capacity to generate massive amounts of data, and to provide a cost-effective method for capturing individual-specific activity patterns at fine resolutions.

Most wrist-worn wearables have been designed with a primary focus of capturing physical activity, especially stepping. However, there is now increasing interest among researchers around their potential for the monitoring of sedentary time (defined as any waking behavior characterized by an energy expenditure ≤ 1.5 metabolic equivalents (METs), while in a sitting, reclining or lying) and sedentary patterns, given evidence linking these exposures to increased risk of cardiovascular diseases, cancer, and mental ill-health [8–10]]. For example, accumulating sedentary time in long uninterrupted bouts has been shown to be associated adversely with glycemic control [11], while interrupting sedentary time lowers postprandial glucose [8]. The proportion of time spent being sedentary is also important because it has been shown to have implications for cardiometabolic health and disease risk, even after adjusting for moderate-to-vigorous-intensity physical activity amount [12].

Validation studies have shown that commercial activity trackers have a reasonable concordance with gold-standard measurements (hand counting) for capturing stepping at faster walking speeds [13], consistent with their primary use. Nevertheless, their measurement of activities of daily living is more problematic, with limited validity for capturing stepping at slower walking speeds [13] and sedentary time [14]. Being wrist-worn, they are also known to have lower sensitivity and specificity for measuring steps when compared to hip-worn and thigh-worn devices [15, 16]. Steps can falsely register during light or sedentary activities involving wrist movement such as folding laundry or playing video games [17]. These spurious detections during activities that do not involve stepping [18, 19] constitute a minority of steps counted and are possibly overlooked as they have limited consequence to some validity statistics for total step counts [20]. However, they are highly relevant to measuring sedentary time, and have a noticeable impact on measuring sedentary bout duration, an issue that has been reported previously [21–23]. To our knowledge, no current algorithms satisfactorily address these issues.

What would be highly desirable are algorithms for consumer-grade devices that can accurately classify physical activities into five widely used categories of differing intensity: sedentary, standing, light physical activity (LPA), moderate physical activities (MPA) and vigorous physical activity (VPA). Notably, in this context, the categories themselves cannot be directly observed. Instead, they would be inferred from parameters reflecting the intensity of the physical activity captured by the wearables, such as heart rate, and either raw acceleration (usually not available), or other device outputs like step counts and estimated metabolic equivalent of task (MET) values. The Hidden semi-Markov Model (HsMM) is a class of stochastic models that can be used to infer the unobserved states that generate the observed variables that reflect those states. Variants of the Hidden Markov Model (HMM) have found prominent applications in meteorology where they have been used to infer the underlying weather states given observed variables such as temperature, air pressure and wind speed [24]. In the context of physical activity, these models have been used to infer the underlying physical activity types (e.g., walking, sitting, and standing) using data from research-grade triaxial accelerometers [25, 26].

Here we describe the development of STEPHEN (STEP and Heart Encoder), a Hidden semi-Markov model to infer the underlying physical activity states using routine output from wrist-worn devices, specifically step counts and heart rate data. We propose to use heart rate data in conjunction with step counts for two reasons. We anticipate that the inclusion of heart rate data would improve detection of sedentary time. Firstly, because physical activities typically increase heart rate, an increase in step counts without significant increase in heart rate may represent misclassified sedentary time involving wrist movements. Secondly, heart rate data may improve the capacity of wrist-worn devices to differentiate sitting from standing events because it increases immediately when standing from sitting/lying position [27].

While STEPHEN infers multiple activity categories, herein we focus on its validity to distinguish sedentary from non-sedentary time and estimating both the proportion of time spent being sedentary and usual sedentary bout duration. These important measures are expected to be poorly captured by current commercial wearables, thus have the potential to be improved. Our focus on sedentary time is also partly driven by the existing data available to us from two studies (OPTIMISE and ALLO-Active), in which participants wore both a common commercial wrist-worn activity tracker (the Fitbit) concurrently with the thigh-worn activPAL, which is highly suitable as a ground truth for sedentary time, but not necessarily for separating between the other physical activity states.

Here, we report our work in developing and evaluating STEPHEN and STEPCODE (STEP encoder) a steps-only based model. We evaluated the accuracy of the models when used to estimate the two key sedentary time metrics (time; usual bout duration) in two external cohorts with different living conditions and using data from different Fitbit devices. The accuracy of the proposed models is compared to proprietary Fitbit software using activPAL as the reference assessment method.

## Methods

### Participants

This study includes participants from two distinct sources: 44 intervention group participants from the OPTIMISE trial [28] and 10 participants from the ALLO-Active trial [29], all of whom wore Fitbit and activPAL monitors (the referent device) concurrently. The OPTIMISE trial focused on reducing sedentary behavior in adults with type 2 diabetes who work in desk-based environments, through provision of a Fitbit, a height-adjustable sit-stand desktop workstation, and behavioral health coaching [28]. Contexts like this, whereby participants are performing activities such as typing that would traditionally be sedentary behaviors, in an upright position (as light activity), are known to pose particular challenges for classifying sedentary time via wrist-worn devices [30]. The ALLO-Active trial involved a very low-active context, where participants spent significant time hospitalised, as the participants were adults with hematologic malignancies who received allogeneic stem cell transplantation. Here the intervention-group participants received a multifaceted activity program designed to increase purposeful aerobic and resistance exercise and reduce sedentary time through replacement with light exercise [29]. The ALLO-Active intervention targeted less extensive behaviour changes by comparison with OPTIMISE.

Human ethics approval for OPTIMISE were obtained from Alfred Health Human Ethics Committee (Melbourne, Australia), The University of Queensland Institutional Human Research Ethics Committee (Brisbane, Australia), and the University of the Sunshine Coast Human Research Ethics Committee (Sunshine Coast, Australia). The OPTIMISE Your Health trial has been registered with the Australian New Zealand Clinical Trials Registry (ANZCTRN12618001159246; date of registration: 07/03/2018). Ethics approval for the ALLO-Active trial was provided by the Alfred Hospital Human Research Ethics Committee, and the trial was registered with the Australian New Zealand Clinical Trials Registry (registration number: ACTRN12619000741189). All participants provided written informed consent.

### Data collection

Both studies used similar protocols for collecting activPAL (PAL technologies, Glasgow, Scotland) data: activPAL4; 24-hour wear protocol; concurrent sleep and wear diary; and devices were attached to the thigh, two-thirds of the way up, along the midline. Devices were set to record data at the standard 30 Hz and were initialised and downloaded with the proprietary software, default settings, default (VANE) algorithm, and exported as events files. OPTIMISE participants wore the activPAL at 0, 3 and 6 months, for 10 days. ALLO-Active trial participants wore the thigh-mounted activPAL at 0, ≈ 1, and ≈ 4 months, for 7 days. ActivPAL measurements from all timepoints were used for validation purposes in this study as long as Fitbit was concurrently worn. OPTIMISE intervention participants received a Fitbit Inspire HR device (Fitbit Inc., San Francisco, CA, USA) while all ALLO-Active trial participants (intervention and usual care) were provided with either a Fitbit Ionic or a Fitbit Versa 2 device (Fitbit, Inc., San Francisco, CA, USA). Both studies encouraged participants to wear the Fitbit continuously throughout the trial period.

### Data processing

Data from both studies were processed identically, limiting the data to when both devices were worn, and the participant was awake (see Details below).

The activPAL events files (VANE) provide a precise record of the start time and duration of each event -each bout of sitting / lying (sedentary) or standing, and each stride (two steps) along with the estimated MET-duration of the event. The default method [31] assigns MET-values of 1.25 to sitting and 1.4 for standing, and estimates METs for stepping as a linear function of cadence (steps/min). The activPAL has strong validity relative to direct observation for classifying sedentary time and transitioning between sedentary and non-sedentary states [32]. This device provides the ground truth for this study. Using SAS 9.4 (SAS Institute, Inc., Cary, NC, USA), based on usual practices in the field [33], time in bed (for sleeping purposes) and non-wear were removed (estimated from a combination of self-report and device movement), and data limited to valid days (≥ 10 h wear while awake with evidence of movement: ≥500 steps/day and < 95% of the day in any one activity) [28]. Automated estimation [34] coupled with visual checking of the automated classification against acceleration using actigrams was used to infer missing values for time in bed. Data were also summarized as amounts of each activity per 1-minute (to permit 1:1 matching of these mixed activity minutes with the Fitbit’s single-activity 1-minute epoch data).

Fitbit data was downloaded using the Fitabase software (Fitabase Small Steps Labs, LLC, San Diego, CA). The Fitbit-generated step counts, heart rate and physical activity intensity were extracted at 1-minute resolution (the smallest resolution available for steps and intensity). Non-wear time (i.e., minutes where the associated heart rate was zero or absent) was excluded along with data between midnight and 5AM, where the person may be wearing the Fitbit and awake but their physical activity patterns may not be representative of the rest of the days. No minimum daily wearing rates criteria was applied to the Fitbit data and no further inspection of the heart rate quality signals was performed.

For validation purpose, the Fitbit data was matched to the activPAL minute-level data and only minutes during which both devices were worn were retained. The activPAL event files were also mapped to the periods during which the participant was wearing the Fitbit and only events where Fitbit was worn 100% of the time were retained. Timestamp matching was performed using in-house R codes that utilize *lubridate* R package [35]. Since activPAL timestamp does not take into account daylight saving shift while Fitbit’s does, we manually shifted back the activPAL timestamp by one hour during time periods where daylight saving was observed. Sedentary behavior metrics were calculated based on these matched data.

### Measures of sedentary time

Because of the differences in the data resolution (1-minute epoch for Fitbit data and continuous time up to the nearest 0.1s for activPAL), the classification of sedentary time is slightly different for the two data types.

For methods that use Fitbit data (Fitbit algorithm, STEPHEN and STEPCODE), the classification took place at each minute. In the case of Fitbit’s proprietary algorithm, Fitabase, a cloud-based Fitbit data management system, was used to extract the predicted physical activity intensity output and its lowest predicted intensity state was designated as sedentary. For STEPHEN and STEPCODE, we obtained the predicted physical activity state for each minute (see Model Development below). For activPAL, the classification rule is simpler whereby all of the time intervals associated with ‘sitting’ events are classified as sedentary.

#### Proportion of time spent being sedentary

For the Fitbit algorithm, STEPHEN and STEPCODE, this was calculated for each participant as the total number of sedentary minutes divided by the total number of minutes when the participant was awake and wearing both devices. For activPAL, this was calculated for each participant as the total time for sedentary (‘sitting’) events divided by the total time for all events.

#### Usual bout duration of sedentary bouts

Sedentary bouts were identified as recorded to the nearest 0.1s duration from the activPAL events files, and as consecutive sedentary minutes for the Fitbit data.

Usual bout duration, defined as the bout duration above and below which half of all *sedentary time* is accrued [36], was calculated for each participant using the methods described in [36] using all of their sedentary bouts across the entire study duration.

**Fig. 1:**
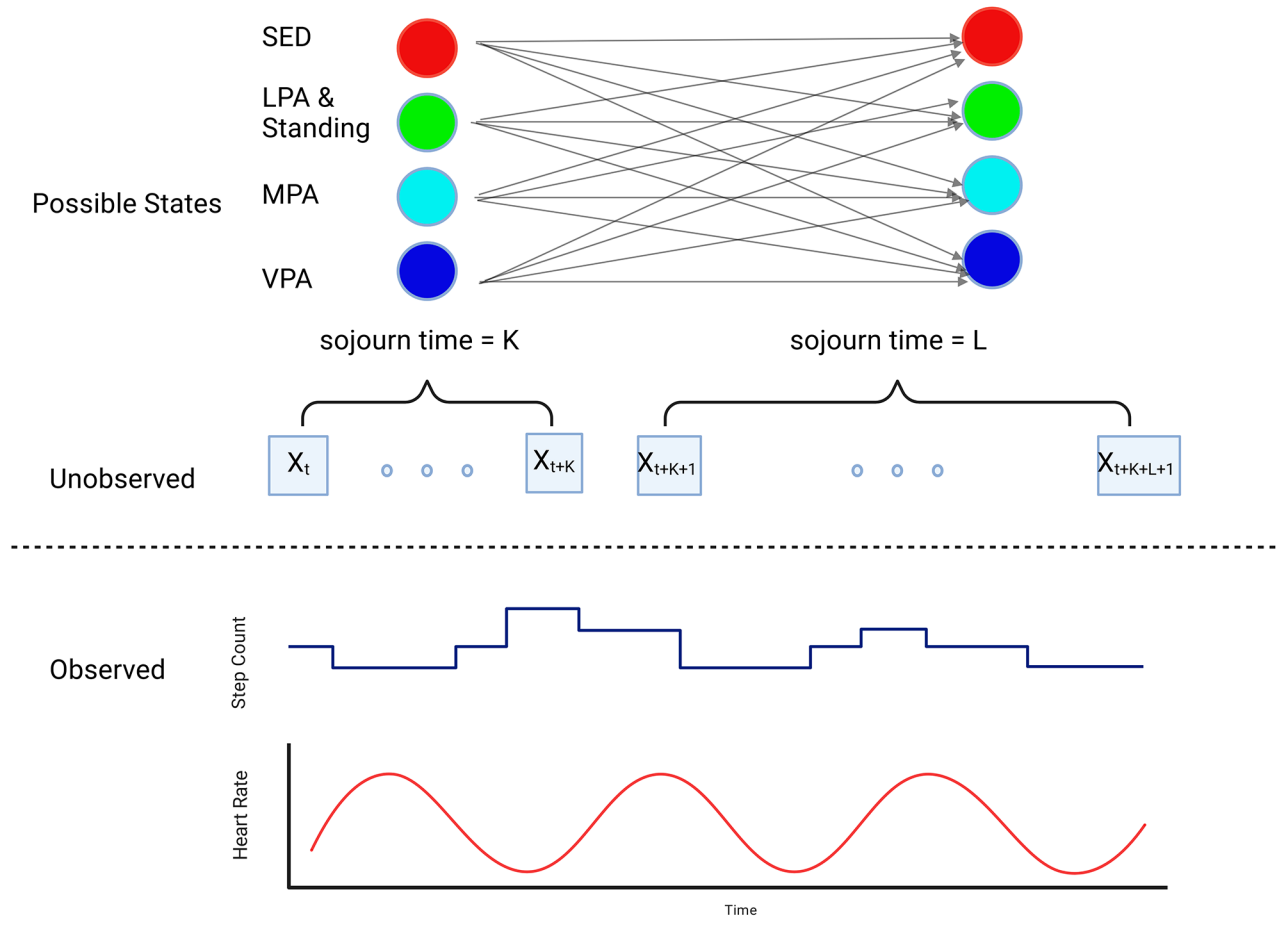
Schematic illustration of the Step and Heart Rate Autoencoder (STEPHEN) Hidden semi-Markov Model. The observed data are step counts and heart rate and individuals can move between the four hidden states representing sedentary behavior, LPA & Standing, MPA and VPA. The time spent in a particular hidden state before moving to another state (sojourn time) is assumed to follow Gamma distribution whose parameters are estimated empirically from the data

### Statistical methods and analyses

#### Model development

We used Fitbit data from 11 OPTIMISE participants to develop 2 models: [1] Hidden semi-Markov Model using step counts and heart rate data (STEPHEN), and [2] Hidden semi-Markov Model using step counts data only (STEPCODE). While the focus of this paper is on validating STEPHEN, STEPCODE was developed to demonstrate that the improved performance of STEPHEN over proprietary algorithm is due to the combination of using heart data in addition to the Hidden semi-Markov model rather than merely due to the Hidden semi-Markov model alone. In both STEPHEN and STEPCODE models, we assume there are K = 4 unobserved physical activity states that manifest as the observed step counts and heart rate data. Without losing generality, we can think of these states as representing sedentary behavior, standing and LPA, MPA and VPA (see Fig. 1). The decision to lump standing with LPA should not affect the validity of the current models for characterising sedentary behaviour because both standings and LPA will be classified as non-sedentary anyway.

Under each state, we assume that the step count and heart rate data are distributed as Negative Binomial (NB) random variables with state-specific parameters. An individual is allowed to move between states, e.g., from the sedentary state to light physical activity with the amount of time (minutes) spent consecutively under a particular state (sojourn) following a Gamma distribution. Compared to the Exponential sojourn distribution for the standard Hidden Markov model, the Gamma sojourn distribution capture persistent sojourn (bouts) under a particular state after the initial transition [37]. This property enables the models to capture prolonged standing and LPA with stable heart rate after the initial heart increase when transitioning from sedentary behavior. The models were estimated separately for each participant, resulting in 11 × 2 = 22 estimated models (11 STEPHEN models and 11 STEPCODE models). We used the mhsmm R package [38] to estimate the model parameters and coded a user-written function to calculate the conditionally independent, bivariate Negative Binomial probability. A more detailed mathematical description of the three models is provided in the Supplementary Methods.

For both STEPHEN and STEPCODE to allow for potential differences in baseline resting heart rate between the individual in the development and validation cohorts, we adjusted the mean heart rate parameters under each state by subtracting a bias term defined as the difference in average heart rate with zero step counts between the individual whose physical activity states we tried to predict and the individual whose data was used to develop the model. We then obtained the predicted physical activity state under each of eleven models (using the Viterbi algorithm). At this stage, the predicted state for each minute is an integer ranging from 1 to 4 and unlabeled. We then labelled these predicted states using the following rules: the predicted states associated with Hidden semi-Markov states with the lowest mean step count and heart rate parameters were labelled as ‘Sedentary’, the remaining predicted states associated with increasing step counts and heart rate parameters were correspondingly labelled as standing/LPA, MPA and VPA. These labelled predicted states were then ensembled at each timepoint using the ‘majority vote’ rule to produce the final, labelled predicted physical activity state.

#### Model validation

An extensive internal validation was performed together with two external validations. Instance-level agreement statistics were not calculated due to the different device resolutions (1 min epoch versus continuous to nearest 0.1s). In lieu of a typical confusion matrix, the amount of time the activPAL recorded as spent sitting, standing and stepping were summarized separately for the minutes that the Fitbit and test methods predicted to be sedentary or non-sedentary. The sedentary time measures (proportion of time spent sedentary and usual bout duration) were compared against the activPAL (the ground truth) via two indicators. The main indicator used was absolute percentage errors (APE), which was summarized per individual and reported as median (MDAPE) with interquartile range (IQR). Mean bias (SD) were visualized via Bland-Altman plots [39] and equivalence between each method and activPAL (Δ_equivalence_ = ± 0.05 for proportion and ± 5 min for usual sedentary bout length) was tested using one sample t-test for equivalence hypothesis testing in R package TOSTER [40] and proportional bias in the Bland-Altman plots were tested using simple linear regression.

#### Internal validation

The internal validation was conducted within the 11 OPTIMISE participants used to develop the model.

#### External validation 1

The first external validation was performed in 33 OPTIMISE participants who were not used for model development, to indicate how well the models are likely to perform in participants with similar demographic characteristics to the participants used to develop the models. The MDAPE for each method was compared via Wilcoxon signed ranks test to determine whether the proposed methods outperformed the proprietary Fitbit method. To investigate whether gender, body mass index (BMI), age and job classification were associated with accuracy, we conducted multiple linear regression with APE as dependent variables.

#### External validation 2

To assess the performance of the models when used to predict sedentary time in participants wearing different brands of Fitbit and with very different physical activity patterns, we tested the models in 10 participants (5 participants wearing Fitbit Ionic and 5 participants wearing Fitbit Versa 2) from the ALLO-Active Trial [29].

All statistical analyses were performed using R 4.2.2.

## Results

### Participant characteristics

The mean (SD) for age and BMI were 52.8 (5.2) years and 35.6 (5.4) kg/m^2^ in the OPTIMISE internal validation sample (*n* = 11) and 52.2 (7.3) years and 33.7 (5.0) kg/m^2^, respectively in the OPTIMISE external validation sample (*n* = 33). ALLO-Active participants were slightly older (57.4 (10.8) years) and leaner (27.2 (5.7) kg/m^2^). Two OPTIMISE internal validation participants (18.2%) and five OPTIMISE external validation participants (15.2%) were on medication that affects heart rates, e.g., beta blockers while none of the ALLO-Active participants were on these medications. Two OPTIMISE external validation participants (6.1%) also have experienced major cardiovascular events.

Among the OPTIMISE participants, the mean (SD) of Fitbit wear time per day was 15.3 (3.0) hours, resulting in the mean (SD) for total Fitbit wear time of 8806.0 (4979.3) hours over the study period, of which 334.3 (174.9) hours was when activPAL concurrently worn. Among the ALLO-Active participants, the mean (SD) of Fitbit wear time per day was 15.0(3.1) hours, resulting in the mean (SD) for total Fitbit wear time of 8508.0 (6504.1) hours over the study period, of which 166.2 (50.1) hours was when activPAL was concurrently worn. Data from the activPAL (referent) during the concurrent wear time indicate the OPTIMSE participants were more active and less sedentary than the ALLO-Active participants, with mean proportion of sedentary time being 0.56(0.12) vs. 0.67(0.11) and mean usual bout duration of 27.3(4.0) vs. 33.3(10.2) minutes.

### Internal validation

STEPHEN, STEPCODE and Fitbit’s proprietary algorithm all showed some degree of agreement with the data from the activPAL (Table 1), with MDAPE (IQR) ranging from 0.14(0.05–0.18) to 0.20(0.10–0.28) for proportion of time spent being sedentary and 0.16(0.03–0.26) to 0.33(0.28–0.39) for usual sedentary bout length (duration). The mean (SD) bias for proportion of time spent being sedentary ranged from − 0.06 (0.11) to 0.13 (0.07) as a proportion, which is equivalent to -57.6 (105.6) to 124.8(67.2) minutes per 16 h awake. The Bland-Altman plots (Supp. Figure 1A-C) show that all methods overestimate the proportion of time being sedentary. In terms of the usual sedentary bout length (duration), STEPHEN had the least bias relative to activPAL(Mean Bias(SD) = 4.40(4.18),Supp. Figure 1E) when compared to Fitbit’s proprietary algorithm (Mean Bias (SD) = -8.68(3.24), Supp. Figure 1D) and STEPCODE (Mean Bias (SD) = -5.14(3.62), Supp. Figure 1F). The 95% limits of agreement were wide for all methods.

**Table 1:** Mean (SD), median (IQR) Absolute percentage errors and Mean (SD) Bias of Sedentary Behavior Metrics against activPAL

Validation	Method	Proportion of Time Spent Sedentary (0–1)				Usual Sedentary Bout Duration (min)			
		Mean (SD)	MDAPE(IQR)	Mean (SD) Bias	*P*-value*	Mean (SD)	MDAPE(IQR)	Mean (SD) Bias	*P*-value*
**Internal** (***n*** = **11)**	activPAL (referent)	0.57(0.11)	**-**	**-**		26.59(5.86)	**-**	**-**	
	Fitbit	0.70 (0.06)	0.20 (0.12–0.28)	0.13(0.07)	0.002	17.91(5.42)	0.33(0.28–0.39)	-8.68(3.24)	0.002
	STEPHEN	0.51 (0.10)	0.14(0.05–0.18)	0.14(0.08)	0.002	31.00(5.08)	0.16(0.03–0.26)	4.40(4.18)	0.939
	STEPCODE	0.69 (0.07)	0.20(0.10–0.28)	0.12/(0.07)	0.006	21.41(6.25)	0.18(0.10–0.29)	-5.14(3.62)	0.367
**External I** (***n*** = **33)**	activPAL (referent)	0.54(0.12)				27.84(5.47)			
	Fitbit	0.68(0.07)	0.23(0.13–0.53)	0.14(0.11)	< 0.001	17.08(4.96)	0.42(0.32–0.48)	-11.93(5.07)	< 0.001
	STEPHEN	0.50(0.09)	0.15(0.06–0.25)	-0.03(0.11)	0.817	33.34(7.69)	0.11(0.06–0.26)	3.91(5.67)	0.857
	STEPCODE	0.67(0.07)	0.20(0.12–0.49)	0.13(0.11)	< 0.001	20.46(6.18)	0.32(0.19–0.39)	-8.90(4.90)	< 0.001
**External II** (***n*** = **10)**	activPAL (referent)	0.67(0.08)				33.26(10.24)			
	Fitbit	0.74(0.09)	0.09(0.05–0.19)	0.07(0.06)	0.186	21.90(4.50)	0.36(0.15–0.48)	-11.35(9.17)	0.029
	STEPHEN	0.77(0.08)	0.13(0.08–0.22)	0.10(0.06)	0.035	33.95(12.95)	0.19(0.03–0.25)	0.70(8.89)	0.957
	STEPCODE	0.74(0.08)	0.09(0.06–0.20)	0.07(0.07)	0.161	26.15 (11.30)	0.30(0.13–0.37)	-7.10(9.55)	0.252
**MDAPE = Median Absolute Percentage Error; IQR = Interquartile Range**									

* Based on testing bias using one-sample t-test for equivalence testing (D_equivalence_ = ± 0.05 for proportion and ± 5 min for usual sedentary bout duration)

The compositions generated from activPAL’s sitting, standing and stepping distributed among the minutes predicted as sedentary and non-sedentary are presented in Table 2. Predicted sedentary time minutes on average consisted of approximately 46s (76.7%) sitting, 13s (21.7%) standing and 1s (1.7%) stepping for all methods. The composition of minutes predicted to be non-sedentary were more variable between the methods (Table 2). Predicted non-sedentary minutes still contained relatively high amounts of sitting, ranging from 11.3s (18.8%) for STEPHEN (which performed best) to 14.0s (23.3%) for STEPCODE. Put simply, most predicted sedentary time (76.7%) was spent sitting, with most of the misclassified activity being standing, while 76.7% (STEPCODE) to 81.2% (STEPHEN) of the time predicted to be not sitting being spent either standing or stepping.

**Table 2:** Mean (SE) of sitting, standing and stepping seconds for Sedentary and Non-sedentary minutes

Validation	Method	Classification	Sitting	Standing	Stepping
Internal (*N* = 11)	Fitbit	Sedentary	46.11(2.36)	12.74(2.18)	1.15(0.25)
	STEPHEN		46.22(2.26)	12.57(2.14)	1.21(0.15)
	STEPCODE		46.45(2.33)	12.55(2.19)	1.00(0.20)
	Fitbit	Non-Sedentary	13.4(0.68)	23.8(1.15)	22.79(0.97)
	STEPHEN		11.31(1.00)	24.72(1.16)	23.98(1.08)
	STEPCODE		14.00(0.91)	23.71(1.19)	22.29(1.00)
External I (*N* = 33)	Fitbit	Sedentary	44.83(1.29)	13.89(1.26)	1.28(0.15)
	STEPHEN		48.76(1.25)	10.36(1.23)	0.88(0.13)
	STEPCODE		45.18(1.29)	13.64(1.26)	1.18(0.14)
	Fitbit	Non-Sedentary	15.27(1.34)	25.06(0.94)	19.68(0.95)
	STEPHEN		13.58(1.41)	24.50(1.09)	21.92(0.75)
	STEPCODE		15.52(1.35)	25.13(0.95)	19.35(0.93)
External II (*N* = 10)	Fitbit	Sedentary	53.36(1.64)	5.64(0.98)	1.00(0.10)
	STEPHEN		52.60(1.03)	6.00(1.00)	1.39(0.12)
	STEPCODE		53.07(1.04)	5.80(1.00)	1.13(0.13)
	Fitbit	Non-Sedentary	16.01(0.98)	25.47(2.43)	18.52(1.76)
	STEPHEN		14.84(1.44)	26.30(2.36)	18.86(1.84)
	STEPCODE		16.38(1.62)	25.50(2.63)	18.12(1.62)

STEPHEN’s non-sedentary minutes contained a higher proportion of either standing or stepping while STEPCODE’s remain very similar to the proprietary algorithm. This suggests that the use of heart rate data improves predictive performance. To better understand how the use of heart rate data results in a better classification of non-sedentary minutes, we examined data from minutes where reclassification from sedentary under Fitbit classification to non-sedentary under STEPHEN occur. More specifically, we focused on a ± 5 min time window around the minutes where the reclassifications occur. The results suggest that except for participant ID 1050, these are the minutes where there is a consistent sudden jump in step counts. It is evident that this sudden jump is consistent because the width of the interval is relatively narrow and either does not include zero or occurs just above zero, (Supp. Figure 2). These consistent changes in the step counts are not accompanied by consistent changes in heart rate (Supp. Figure 3). We interpret this as evidence that the sudden apparent increase in steps is very likely due to wrist movements that are not associated with physical activities, and STEPHEN managed to successfully reclassify Fitbit anomalous non-sedentary minutes as sedentary.

**Fig. 2:**
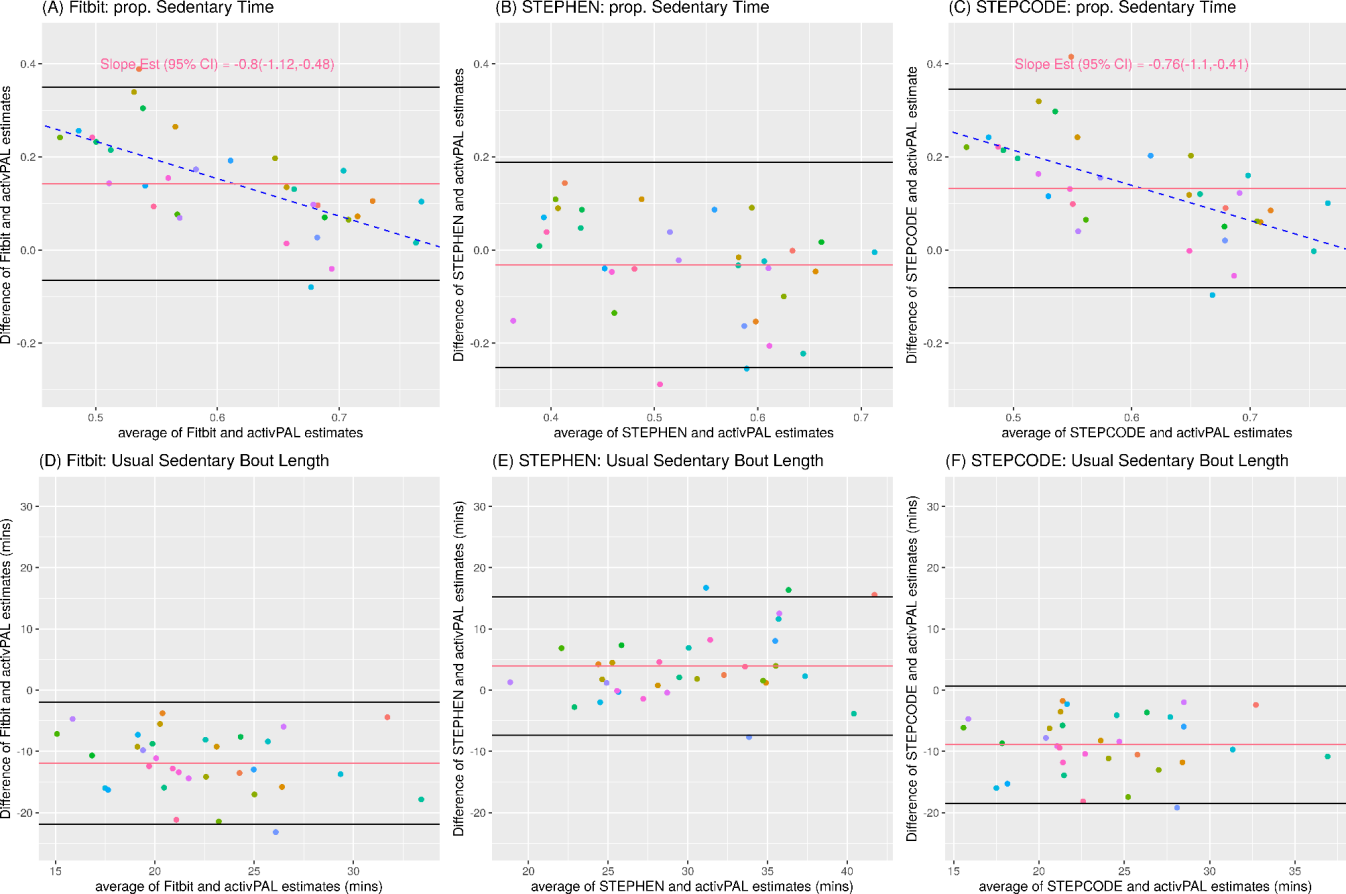
External Validation Using *N* = 33 OPTIMISE Participants. (**A**)-(**C**) Bland-Altman Plots of Proportion of Time Spent Being Sedentary according to: (**A**) Fitbit, (**B**) STEPHEN, (**C**) STEPCODE. (**D**)-(**F**) Bland-Altman Plots of Usual Sedentary Bout Length (Duration) according to: (**D**) Fitbit, (**E**) STEPHEN, (**F**) STEPCODE. In all plots the center line indicates the mean bias and the top and bottom lines indicate the limits of agreements (mean ± 1.96 SD). Statistically significant proportional bias was shown with 95% confidence intervals of the slope estimate

**Fig. 3:**
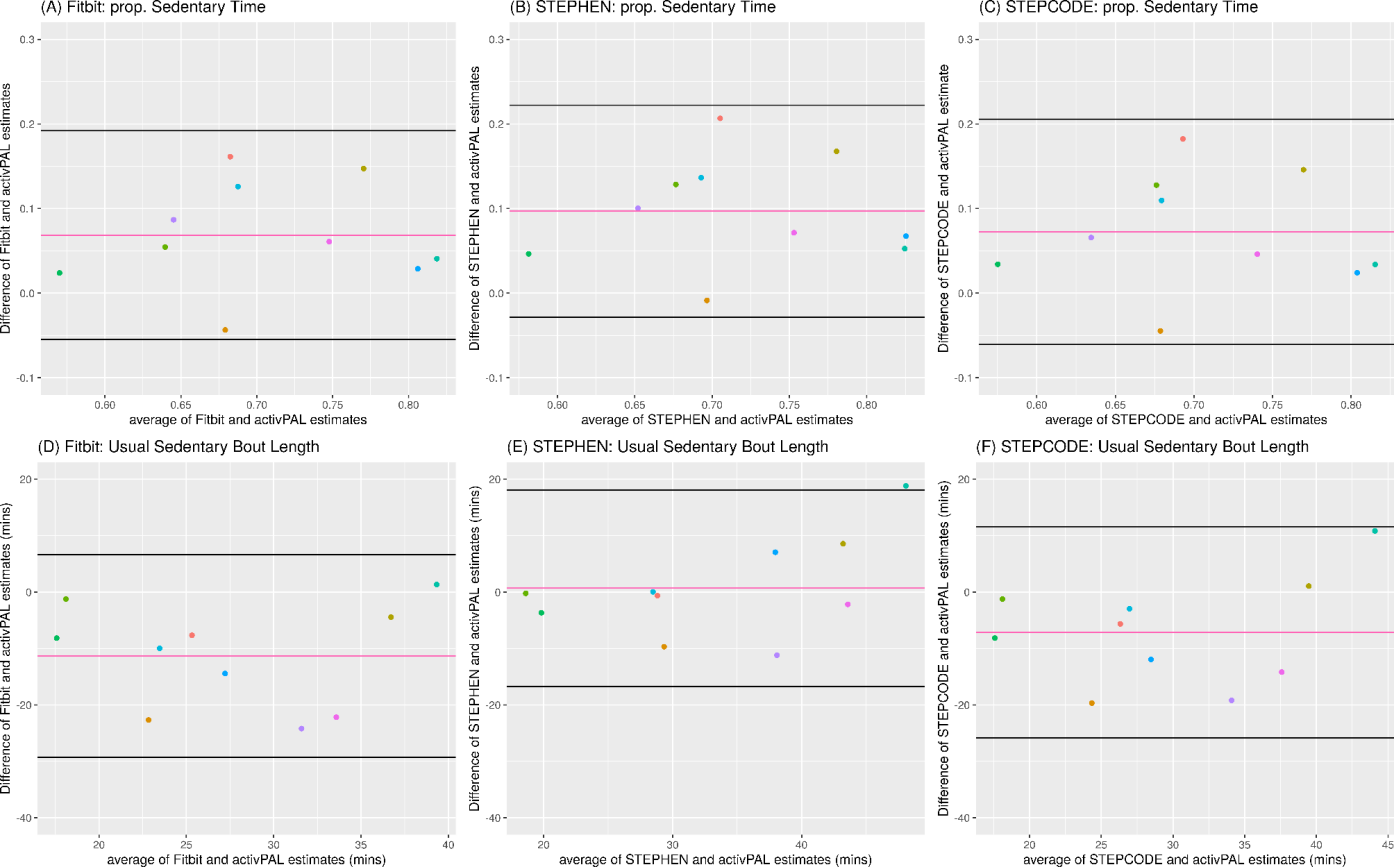
External Validation Using *N* = 10 ALLO-Active Participants. (**A**)-(**C**) Bland-Altman Plots of Proportion of Time Spent Being Sedentary according to: (**A**) Fitbit, (**B**) STEPHEN, (**C**) STEPCODE. (**D**)-(**F**) Bland-Altman Plots of Usual Sedentary Bout Length (Duration) according to: (**D**) Fitbit, (**E**) STEPHEN, (**F**) STECODE. In all plots the center line indicates the mean bias and the top and bottom lines indicate the limits of agreements (mean ± 1.96 SD). Statistically significant proportional bias was shown with 95% confidence intervals of the slope estimate

### External validation 1

In the OPTIMISE external validation sample, the accuracy for proportion of time spent being sedentary and usual bout duration was the best for STEPHEN. The MDAPE (IQR) for proportion of time being sedentary was lowest for STEPHEN, at 0.15(0.06–0.25), followed by 0.20(0.12–0.49) for STEPCODE and 0.23(0.13–0.53) for Fitbit (Table 1). Both STEPHEN and STEPCODE differed significantly in MDAPE relative to the Fitbit (both *p* < 0.001). The same was seen for usual sedentary bout duration, where the MDAPE (IQR) was 0.11(0.06–0.26) for STEPHEN, 0.32(0.19–0.39) for STEPCODE and 0.42(0.32–0.48) for Fitbit and both methods differed significantly in MDAPE relative to the Fitbit (both *p* < 0.001).

STEPHEN also resulted in the smallest mean bias for both proportion of time spent being sedentary (Bias Mean(SD) = -0.03 (0.11), equivalent to 28.8 min/16 hours awake) and usual sedentary bout duration (Bias Mean(SD) = 3.91(5.76) minutes, Table 1). Among the three methods (Fitbit, STEPHEN and STEPCODE), only STEPHEN demonstrated equivalence with activPAL (*p*-values = 0.817 and 0.857 for proportion of time spent being sedentary and usual bout duration, Table 1). In contrast, both Fitbit and STEPCODE have the equivalence hypothesis rejected for both metrics (*p*-values < 0.001, Table 1).

The Bland-Altman plots revealed that the 95% limits of agreement were rather wide for all methods (Fig. 2) and there were statistically significant proportional biases for the estimate of proportion of sedentary time under Fitbit’s algorithm (*p*-value < 0.001, Fig. 2A) and STEPCODE (*p*-value < 0.001, Fig. 2C) but not for STEPHEN (*p*-value = 0.098, Fig. 2B). The Bland-Altman plots for usual sedentary bout did not exhibit proportional bias (all *p*-values > 0.10, Fig. 2D-F).

Among minutes predicted as sedentary time, the compositions of activPAL’s sitting, standing and stepping (Table 2) were approximately 45s (75.0%), 14s (23.3%) and 1s (1.7%) for Fitbit’s algorithm and STEPCODE, while STEPHEN’s sedentary minutes contains more sitting, at approximately 49s (81.3%) and less standing, at approximately 10s (16.7%). Among minutes predicted as non-sedentary, the compositions for Fitbit’s algorithms and STEPCODE were approximately 15s (25.0%) sitting, 25s (41.7%) standing and 20s (33.3%) stepping. STEPHEN’s non-sedentary minutes contains slightly less sitting at approximately 14s (23.3%) and more standing (25s, 41.7%).

The accuracy of STEPHEN, as well as STEPCODE and Fitbit’s proprietary algorithm appeared to be quite robust across males and females (Table 3), but showed dependence on age for proportion of time spent being sedentary and on BMI for usual sedentary bouts (Table 3). Although in both cases the effect sizes are rather small. On the other hand, the APE appears to be much smaller (accuracy is much better) for those in Managers/Administrators job category (Table 3), although these are not statistically significant.

**Table 3:** Regression coefficients and 95% confidence intervals for Investigating Association between Demographic Factors and accuracy as measured using Absolute percentage error (APE)

		Proportion of Time Spent Sedentary					
		Fitbit		STEPHEN		STEPCODE	
Variables		Coef.	95% CI	Coef.	95% CI	Coef.	95% CI
Gender	Female	ref		ref		ref	
	Male	0.076	(-0.096-0.247)	-0.061	(-0.171-0.051)	0.073	(-0.092-0.238)
Body Mass Index (BMI)		-0.016	(-0.031–0.001)	-0.006	(-0.015-0.004)	-0.014	(-0.028-0.001)
Age (years)		0.011	(0.001–0.020)	-0.002	(-0.008-0.004)	0.011	(0.001–0.019)
Job Category	Associate Professionals	ref		ref		ref	
	Clerical/Sales/Service Workers	-0.032	(-0.259-0.194)	-0.019	(-0.166-0.128)	-0.021	(-0.239-0.197)
	Managers and Administrators	-0.151	(-0.392-0.092)	-0.083	(-0.240-0.074)	-0.121	(-0.353-0.112)
	Professionals	-0.027	(-0.219-0.165)	-0.078	(-0.203-0.046)	-0.013	(-0.198-0.172)
		Usual Sedentary Bout					
		Fitbit		STEPHEN		STEPCODE	
		Coef.	95% CI	Coef.	95% CI	Coef.	95% CI
Gender	Female	ref		ref		Ref	
	Male	-0.127	(-0.244–0.011)	0.014	(-0.114-0.141)	-0.128	(-0.278-0.021)
Body Mass Index (BMI)		-0.011	(-0.021–0.001)	0.012	(0.001–0.024)	-0.011	(-0.025-0.002)
Age (years)		0.003	(-0.004-0.009)	0	(-0.007-0.007)	0.002	(-0.006-0.010)
Job Category	Associate Professionals	ref		ref		ref	
	Clerical/Sales/Service Workers	-0.054	(-0.208-0.1)	-0.026	(-0.194-0.143)	-0.071	(-0.268-0.127)
	Managers and Administrators	-0.117	(-0.281-0.047)	-0.068	(-0.247-0.112)	-0.128	(-0.339-0.083)
	Professionals	-0.061	(-0.191-0.069)	0.011	(-0.133-0.153)	-0.062	(-0.23-0.106)

Similar to the observed proportional bias in Fitbit’s and STEPCODE’s estimate of proportion of time spent being sedentary, the APE was also significantly associated with the amount recorded by the activPAL (regression coefficient = -2.03, 95% CI = [-2.43 ; -1.63], *p*-value < 0.001 for Fitbit, -1.97, 95% CI = [-2.33 ; -1.61], *p*-value < 0.001 for STEPCODE and − 0.33 [-0.77;0.12] for STEPHEN).

#### External validation 2

The second external validation in the more sedentary, less active population (ALLO-Active) showed slightly different results to those seen in the OPTIMISE external validation (Table 1). As in the OPTIMISE external validation, the STEPHEN model had the highest accuracy for estimating usual sedentary bout duration with MDAPE(IQR) = 0.19(0.03–0.25), followed by STEPCODE with MDAPE(IQR) = 0.30(0.13–0.37) and Fitbit’s algorithm with MDAPE(IQR) = 0.36(0.15–0.48). However, the accuracy for proportion of time being sedentary by STEPHEN was slightly worse than Fitbit’s (MDAPE(IQR) = 0.13(0.08–0.22) vs. 0.09(0.05–0.19)). On the other hand, STEPCODE had similar accuracy to Fitbit for this metric with MDAPE(IQR) = 0.09(0.06–0.20). At the instance level, all methods have predicted sedentary minutes consisting of approximately 53s (88.3%) sitting, 6s (10.0%) standing and 1s (1.7%) stepping. For non-sedentary minutes, STEPHEN had approximately 15s (25.0%) sitting, 26s (43.4%) standing and 19s (31.7%) stepping, while Fitbit and STEPCODE are quite similar with approximately 16s (26.7%) sitting, 26s (43.4%) standing and 18s (30.0%) stepping.

Consistent with the MDAPE results, the mean biases (Table 1) showed slightly less overestimation of sedentary time proportion for both Fitbit and STEPCODE than for STEPHEN (Mean (SD) Bias = 0.10(0.06)), and a much lesser mean (SD) bias in usual bout duration for STEPHEN with 0.70(8.89) minutes compared with STEPCODE with − 7.10(9.55) minutes and Fitbit with − 11.35(9.17) minutes. All methods demonstrated equivalence with activPAL for both metrics (all *p*-values > 0.05, Table 1).

Interestingly, proportional biases were not as apparent in the ALLO-Active population as they had been in the OPTIMISE validation sample (all *p*-values > 0.10, Fig. 3).

## Discussion

We have developed STEPHEN, a Hidden semi-Markov Model for classifying activity from the routine output (minute-level steps and heart rate) of commercially available wrist-worn wearable devices and tested its capacity to detect sedentary time. STEPHEN provided strong sedentary time classification both at a population-level and at an instance-level within an individual. Collectively, the classification improvements were seen in the internal validation, an external validation using OPTIMISE participants, and to a slightly lesser extent, in a very different external population (who by comparison with participants used to train the model were more sedentary, less active, and did not have access to sit-stand workstations). STEPHEN improved sedentary time classification through effective use of heart rate data for removing segments associated with wrist-only movements that result in high step counts but are unaccompanied by proportional change in heart rate. STEPHEN’s sedentary minutes contains a higher proportion of sitting time (as reflected by activPAL), while its non-sedentary minutes contains less sitting and higher proportion of standing or stepping. This finding demonstrates that overall, the use of heart rate data results in better detection of postural change. However, we acknowledge that STEPHEN’s classification is still far from perfect. In particular, there are still sizeable sitting events among minutes classified as non-sedentary. This misclassification is directly related to the fact that increases in heart rate without postural change could occur due to events such as stress, anger and underlying health conditions such as tachycardia. Indeed, this highlights the challenges of accurate detection of postural change ©using wrist-worn devices. Recently, Straczkiewcz et al. developed a novel method that can be used to improve detection of postural change using data from wrist-worn devices [30]. However, their approach relies on raw accelerometer data unavailable to consumers and researchers without the proprietary Application Programming Interface (API), hence potentially limiting its utility.

For over a decade it has been demonstrated that for research-grade devices such as the Actiheart that collect heartrate in a cumbersome way on the chest, energy expenditure can be captured more accurately via combined heart rate and accelerometry than by accelerometery alone or heart rate alone [41]. Our experience with STEPHEN further supports the importance of measuring heart rate data with wearable devices for its utility in distinguishing lower energy expenditure activities such as sedentary behaviors in addition to measuring physical activ–ty - even when using only simple measures taken at the wrist on commercial wearables. The benefit of using heart rate may have particular relevance in settings that involve using sit-stand workstations to perform activities with the same wrist movements sometimes in a seated position (sedentary), and sometimes in an upright position (non-sedentary). Sit-stand workstations are an important context for research applications, as they are a highly effective means of reducing sedentary time especially when part of multicomponent interventions [42].

We developed STEPHEN using data from a particular type of Fitbit device (Fitbit Inspire HR) but the model underlying STEPHEN only requires step counts and heart rate data. Thus, in theory STEPHEN can be trained using data from other wrist-worn devices that produce step counts and heart rate data as part of their output. Its performance, however, would depend on the reliability of the heart rate data. There are several factors that have been shown to have effects on heart rate accuracy, and/or step counts, and other data generated from acceleration, such as wrist circumference, wrist placement, tightness of device and dominant vs. non-dominant hand use [43]. A recent meta-analysis also showed that devices with optical heart rate sensors are generally more accurate for estimating energy expenditure [44]. In the future, we plan to add more features to our models, so that information on these factors, when available, can be used to improve the sedentary time estimates. Incorporating heart-rate variability will be viable in the not too distance future when collection of higher-resolution heart rate data becomes the norm and may also help with classification. Access to raw acceleration data – which are usually not readily available to consumers from commercial wearables without access to proprietary API and possibly collaboration with the relevant industry partner – would also open further possibilities for improved classification based upon acceleration (with or without heart rate). The highly detailed raw data, which typically are available from research-grade wearables (that often lack heart rate data), offers possibilities of capturing a wider range of inputs, including position [45], and the application of machine learning methods to improve classification of sedentary time as well as physical activity [46].

## Conclusions

Overall, the findings demonstrate that STEPHEN provides a better, viable alternative to the proprietary algorithm for estimating sedentary times using wrist-worn devices, especially when prior data are available to train the model for specific living conditions. Importantly, the accuracy of STEPHEN was reasonably stable across individuals and characteristics and, in contrast to Fitbit’s proprietary algorithm, does not exhibit proportionality bias. The methods are also timely given that methods for estimating sedentary time using wrist-worn devices lag behind similar methods for hip or thigh-worn devices [47, 48]. We implemented our methods via an open access R package (https://github.com/limfuxing/STEPHEN/) and users can opt to use the built-in trained models or train their models from scratch to suit specific living conditions. Retraining STEPHEN for specific populations is good practice and recommended. Nonetheless, when this cannot be performed for practical reasons, the external validation results support STEPHEN as being preferable to the proprietary output from the Fitbit for measuring sedentary time accumulation and is likely also to be superior for estimating proportion of time spent being sedentary, particularly for intervention-trial contexts.

STEPHEN clearly improves upon the proprietary algorithm for sedentary time and provides initial indications it could be preferred over the proprietary algorithm when data from higher quality devices are unavailable. However, the findings here should not be taken as indicating this approach for consumer wearables is preferable to estimating sedentary time using devices that can produce high quality posture-driven sedentary measures such as activPAL. We have not compared the performance of STEPHEN to research grade wrist-worn devices such as ActiGraph. But it is worth noting that these research grade wrist-worn devices, due to wrist placement are also sensitive to upper extremity movement [45] and hence could also be affected by anomalous step counts due to activities that involve wrist-only movement.

The key strengths of the study were the inclusion of two markedly different study populations over a reasonably long period of observation. Limitations of using the study results (and the algorithm) are firstly that the user will need to apply a further method for separating out time in bed or sleep from sedentary time. STEPHEN (like Fitbit’s proprietary algorithm) does not separate out time in bed for sleep (or sleep) from sedentary time, however, the validation process was limited to data during waking time when both devices were worn. Secondly, while STEPHEN has the potential to estimate physical activities, we have only validated its performance for estimating sedentary behavior. Future extension of STEPHEN for estimating physical activities would need to consider modelling the LPA and standing states separately.

## Electronic supplementary material

Below is the link to the electronic supplementary material.

Supplementary Material 1

Supplementary Material 2

Supplementary Material 3

Supplementary Material 4

## Data Availability

No datasets were generated or analysed during the current study.

## Abbreviations

APE: Absolute percentage errors
API: Application programming interface
BMI: Body mass index
CI: Confidence interval
HMM: Hidden Markov Model
HsMM: Hidden semi Markov Model
HR: Heart rate
IQR: Inter quartile range
LPA: Light physical activity
MAD: Mean absolute deviation
MDAPE: Median absolute percentage error
MET: Metabolic equivalent of task
MPA: Moderate physical activity
NB: Negative Binomial
SD: Standard deviation
STEPCODE: STEP enCODEr
STEPHEN: STEP and Heart ENcoder
VPA: Vigorous physical activity
