# Supplementary material for: Detection of sedentary time and bouts using consumer-grade wrist-worn devices: a hidden semi-Markov model
Source: BMC Med Res Methodol. 2024 Sep 30;24:222. doi: 10.1186/s12874-024-02311-5 (PMC11440759; doi:10.1186/s12874-024-02311-5)
Supplement: Supplementary file 1 — Supplementary Material 1 [file 12874_2024_2311_MOESM1_ESM.pdf]

Supplementary Methods of "Detection of sedentary time and bouts using consumer-grade wrist-worn device: A Hidden semi-Markov model"

Salim et al.

Method

The Hidden semi-Markov Model (HsMM) and the Hidden Markov Model (HMM) Using Step counts and Heart Rate data

We assume there are $K = 4$ states of physical activity, corresponding to sedentary behaviour (SB), light PA, moderate PA and vigorous PA. The states are not directly observed and instead we observe the step counts and heart rate data as manifestation of the states. At time t, conditional on the unobserved state $X_t = k$, the step counts (Y_{1t}) and heart rate (Y_{2t}) are distributed as independent Negative Binomial random variables,

$$\begin{aligned} Y_{1t} \mid X_t = k &\sim \text{NB}(\mu_1^k, \psi_1^k) \\ Y_{2t} \mid X_t = k &\sim \text{NB}(\mu_2^k, \psi_2^k) \\ P(Y_{1t}, Y_{2t} \mid X_t = k) &= P(Y_{1t} \mid X_t = k)P(Y_{2t} \mid X_t = k) \end{aligned}$$

What differentiates the Hidden semi Markov model (HSMM) from the standard HMM is the assumption on the distribution of sojourn time, i.e., time spent on a particular state before moving to another state. HMM implicitly assumes that the sojourn time follows exponential distribution. HSMM relaxes this assumption and allows the sojourn time to be explicitly specified. Here, we use Gamma distribution to model the sojourn time. Specifically, the sojourn time for state k follows Gamma distribution with scale parameter α_k and shape parameter β_k, so the mean sojourn time for state k is $\alpha_k \beta_k$.

HsMM Step model

To demonstrate the benefit of utilizing heart rate data for estimating sedentary behaviour, we also fitted model with only step count as the observed random variable and compare the performance of this model to the Step-HR model above and fitbit classification.

Under this model, as before we assume there are K states of physical activity. The states are not directly observed and instead we observe the step counts and heart rate data as manifestation of the states. At time t, conditional on the unobserved state $X_t = k$, the step counts (Y_{1t}) are distributed as independent Negative Binomial random variable,

$$Y_{1t} \mid X_t = k \sim \text{NB}(\mu_1^k, \psi_1^k)$$
