# Supplementary figures and images for: Detection of sedentary time and bouts using consumer-grade wrist-worn devices: a hidden semi-Markov model
Source: BMC Med Res Methodol. 2024 Sep 30;24:222. doi: 10.1186/s12874-024-02311-5 (PMC11440759; doi:10.1186/s12874-024-02311-5)

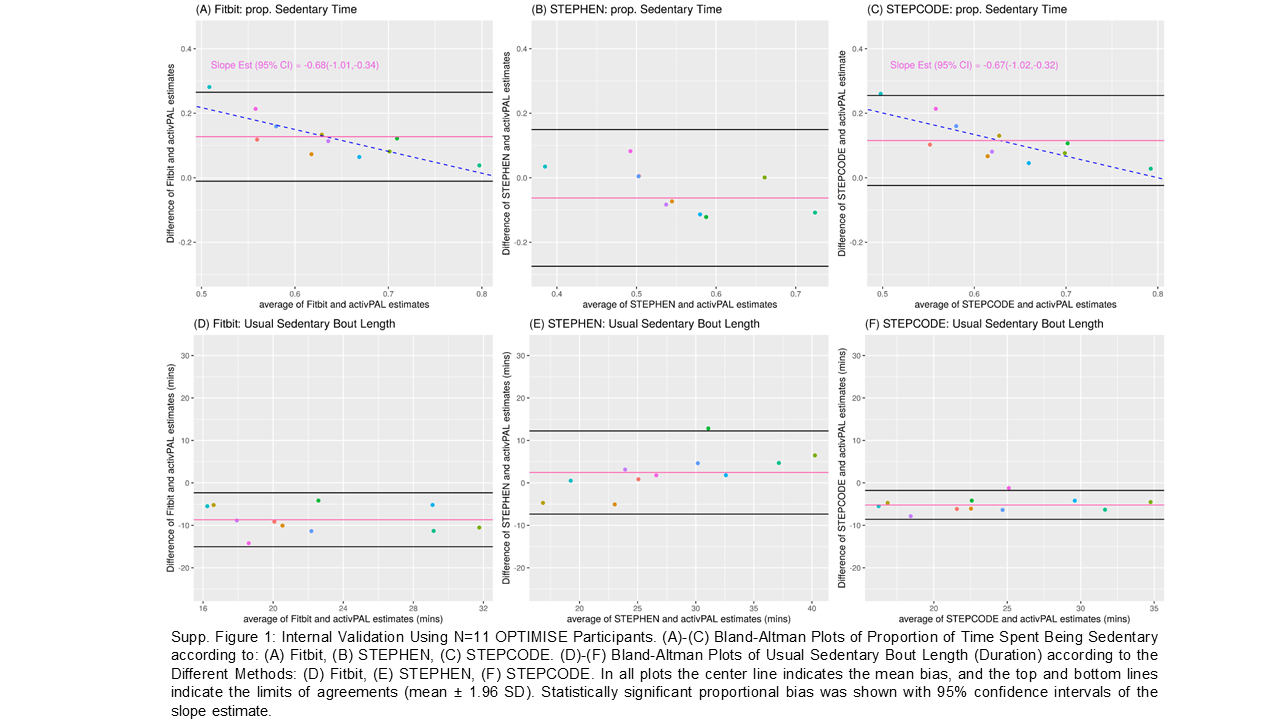

Supplement: Supplementary file 2 — Supplementary Material 2 [file 12874_2024_2311_MOESM2_ESM.tif]

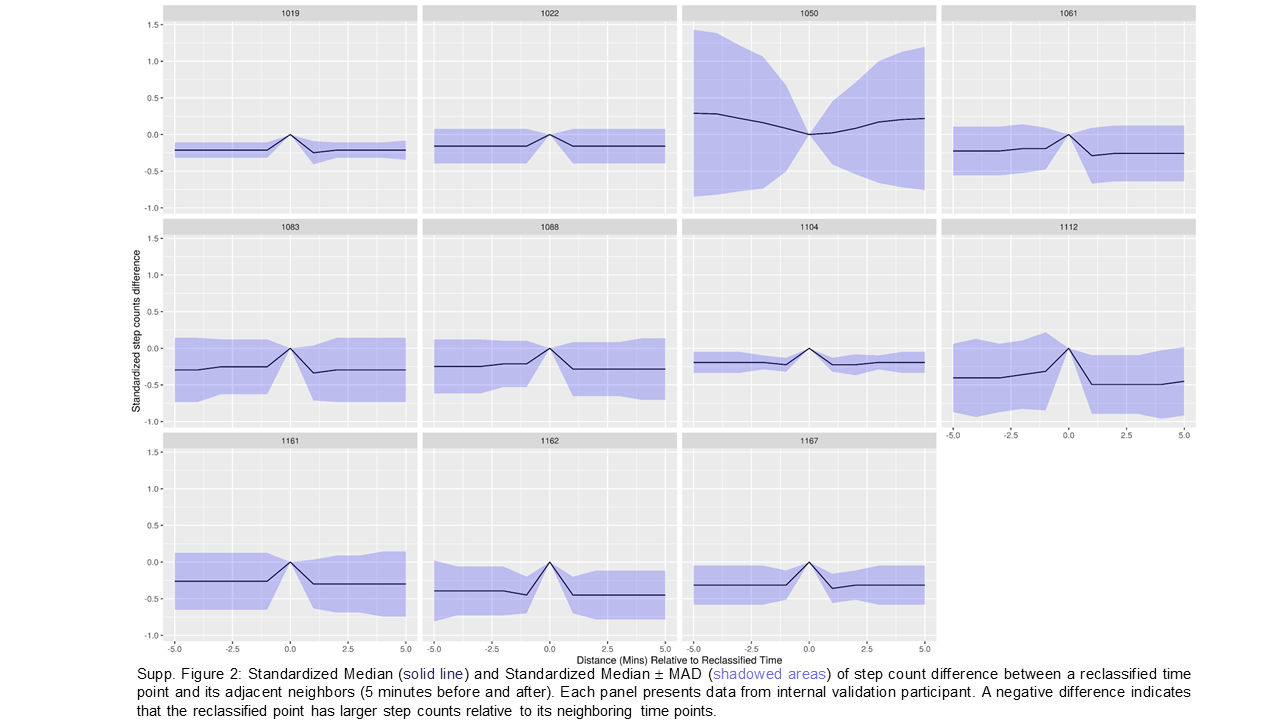

Supplement: Supplementary file 3 — Supplementary Material 3 [file 12874_2024_2311_MOESM3_ESM.tif]

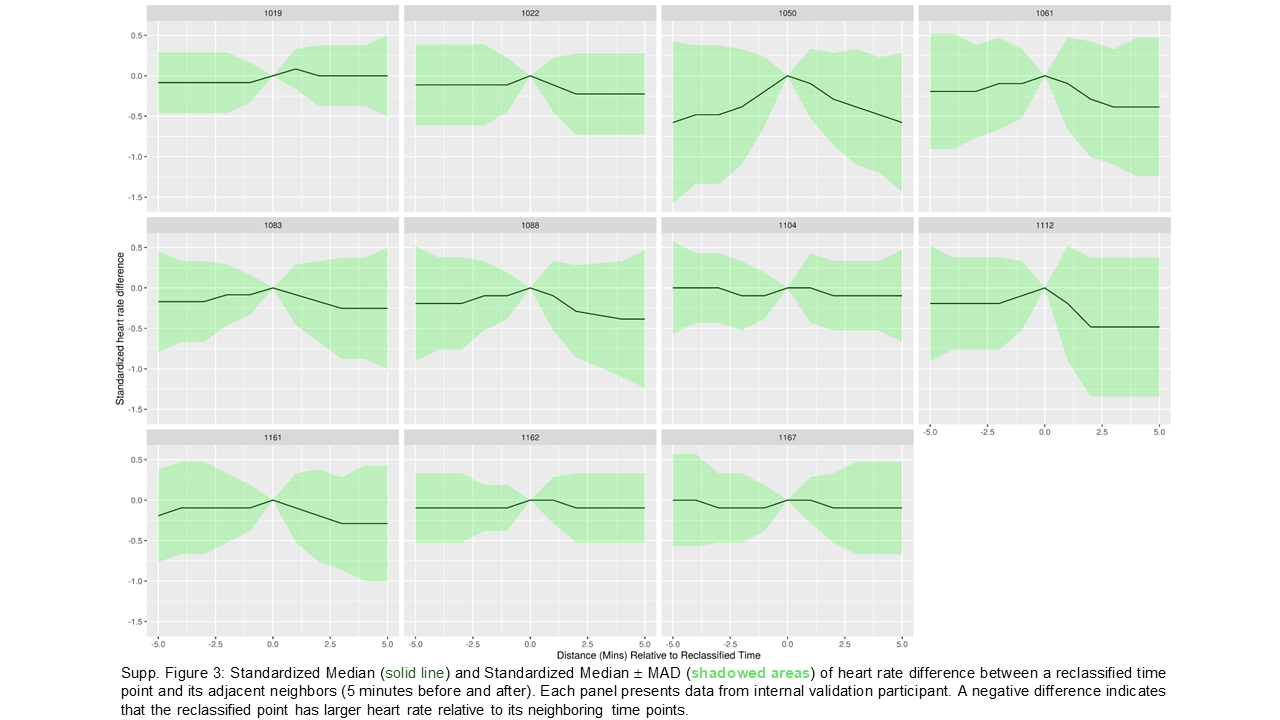

Supplement: Supplementary file 4 — Supplementary Material 4 [file 12874_2024_2311_MOESM4_ESM.tif]
